# Phase-Incremented Steady-State Free Precession as an Alternate Route to High-Resolution NMR

**DOI:** 10.1021/jacs.3c12954

**Published:** 2024-01-31

**Authors:** Tian He, Yuval Zur, Elton T. Montrazi, Lucio Frydman

**Affiliations:** # Department of Chemical and Biological Physics, Weizmann Institute, 7610001 Rehovot, Israel; $ Department of Chemistry, Zhejiang University, Hangzhou 310058, China; † Insightec Ltd, 3903203 Tirat Carmel, Israel

## Abstract

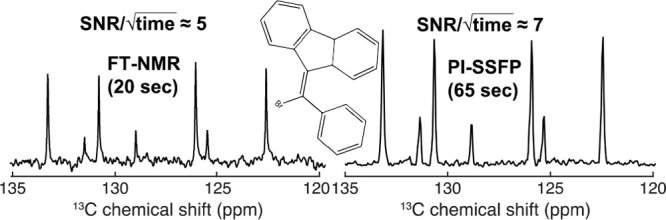

Pulsed Fourier transform nuclear magnetic resonance (FT-NMR) has reigned supreme in high-resolution, high-field spectroscopy—particularly when targeting complex liquid-state samples involving multiple sharp peaks spread over large spectral bandwidths. It is known, however, that if spectral resolution is not a must, the FT-based approach is not necessarily the optimal route for maximizing NMR sensitivity: if *T*_2_ ≈ *T*_1_, as often found in solutions, Carr’s steady-state free-precession (SSFP) approach can in principle provide a superior signal-to-noise ratio per √(acquisition_time) (SNR_t_). A rapid train of pulses will then lead to a transverse component that reaches up to 50% of the thermal equilibrium magnetization, provided that pulses are applied at repetition times TR ≪ *T*_2_, *T*_1_, and that a single suitable offset is involved. It is generally assumed that having to deal with multiple chemical shifts deprives SSFP from its advantages. The present study revisits this assumption by introducing an approach whereby arbitrarily short SSFP-derived free induction decays (FIDs) can deliver high-resolution spectra, without suffering from peak broadenings or phase distortions. To achieve discrimination among nearby frequencies, signals arising from a series of regularly phase-increased excitation pulses are collected. Given SSFP’s amplitude and phase sensitivity to the spins’ offset, this enables the resolution of sites according to their chemical shift position. In addition, the extreme fold-over associated with SSFP acquisitions is dealt with by a customized discrete FT of the interpulse time-domain signal. Solution-state ^13^C NMR spectra which compare well with FT-NMR data in terms of sensitivity, bandwidth, and resolution can then be obtained.

Although the first decades of magnetic resonance witnessed alternative signal acquisition protocols,^1−4^ the last 50 years firmly set FT-NMR as *the* route for collecting 1D NMR spectra.^5^ The acquisition of sufficiently long FIDs resolving closely spaced lines at unknown positions while covering arbitrary bandwidths without penalties, provided FT-NMR with the SNR_t_ needed to tackle low-abundance, low-γ species like ^13^C.^6,7^ Achieving quality line shapes demands letting a FID decay down into noise-like levels, and although early studies considered the possibility of signal averaging under other conditions,^4^ these were abandoned in favor of collecting long FIDs using the Ernst angle for optimal sensitivity.^8,9^ This in turn left outside the realm of high-resolution NMR early SSFP propositions,^10^ which for *T*_2_ ≈ *T*_1_ conditions could actually deliver high SNR_t_ by applying a train of equidistant RF pulses of constant flip angle α spaced by repetition times TR ≪ *T*_2_.^11−15^ In liquid NMR, this choice is unappealing as spectra will then be deprived from resolution; in MRI, where only water is of interest and rapid acquisitions are of essence, the ensuing SNR_t_ gains see widespread applications.^11−13^ Even in MRI, however, SSFP is not universally used: the sequence suffers from a high sensitivity to offsets, leading to signal losses and the appearance of dark bands.^11−16^ On the other hand, this dependency on offset also means that, somewhere, SSFP has the capability to deliver chemical shift discrimination. The present study presents a route to achieve this discrimination for arbitrarily short TRs, and explores the performance of the ensuing approach to 1D ^13^C NMR acquisitions.

It is worth highlighting SSFP’s main features, which we do following Zur et al. and Vasanawala et al.^15−17^ Upon applying an equidistant train of α pulses with phase θ separated by times TR ≪ *T*_2_ (Figure 1A), a transverse steady-state magnetization will—after an initial transient—be established. The ensuing FID will depend on α as well as on a peak’s chemical shift ω; for a single-site this can be written as
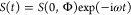
1awhere *S*(*t*) is the signal at any 0 ≤ *t* ≤ TR time between consecutive pulses, and Φ = ω·TR is the phase accrued in-between pulses, defining the signal *S*(0, Φ) right before any pulse in the train. Given SSFP’s time periodicity, *S*(0, Φ) will also be periodic over angular frequency intervals that are multiples of 2π/TR.^11−15^ In other words
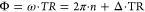
1bwhere *n* is an integer and −π/TR ≤ Δ ≤ + π/TR. Focusing first only on isochromats within this interval Δ (Figure 1B), the steady-state signal in eq 1a can also be written as^16^
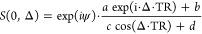
2awhere  is the phase imparted by the RF pulse, and the *a*, *b*, *c*, *d* coefficients are functions of *E*_1_ = exp(−TR/*T*_1_), *E*_2_ = exp(−TR/*T*_2_), and α—but independent of Δ. Given *S*(0, Δ)’s 2π/TR periodicity, this SSFP signal can also be expanded as a Fourier series
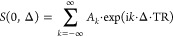
2bwhere the *A*_*k*_ Fourier components can be analytically calculated, and depend on α, *E*_2_, and *E*_1_.^16^ The present study relies on these *A*_*k*_ coefficients and on *S*’s amplitude dependence on Δ, to separate the contributions arising from different chemical sites within each ±π/TR interval.

**Figure 1:**
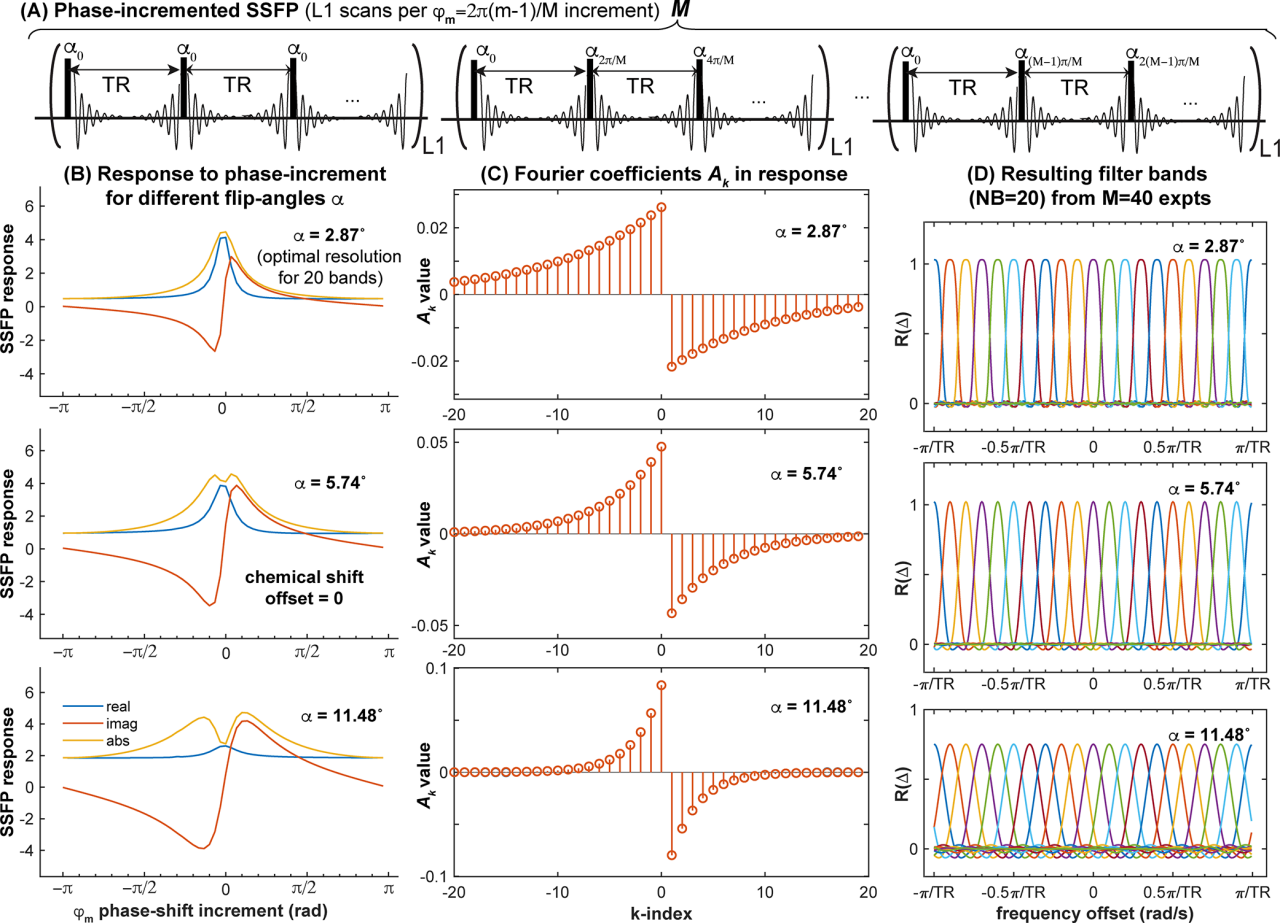
(A) Schematic pulse sequence describing the phase-incremented SSFP approach discussed in this work, involving a train of pulses of flip angle α spaced by a time TR, that are phase-incremented over *M* consecutive experiments where phase increments take progressively larger values ϕ_*m*_ = 2π·*m*/*M* (0 ≤ *m* ≤ *M* – 1). For each phase increment, L1 identical scans are averaged for SNR_t_ enhancement. The cartoon assumes a very strong *T*_2_* effect; in actuality the FID is collected within a TR ≪ *T*_2_ and is nearly flat. (B) Single-site SSFP response vs flip angle and relative phase increment ϕ_*m*_, assuming a constant receiver phase of zero. A similar response would arise from a constant phase ϕ, as a function of the site’s offset. (C) Fourier coefficients making up the SSFP response in panel B; notice their drop with increasing flip angles α. (D) Filters arising from eq 7 for *M* = 40 different phase-incremented experiments; notice the increased widths and wiggles arising in these filters—that will eventually become the point-spread functions of the peaks to be separated—with increasing flip angles.

SSFP’s offset dependence and fold-over patterns have been considered this technique’s main drawbacks. Schwenk proposed dealing with these by Quadriga spectroscopy,^18^ while in MRI experiments are often collected at different offsets to avoid artifacts.^17,19^ This work exploits these shift-compensation ideas–not to erase SSFP’s offset dependence, but rather to sharpen it, and thereby introduce resolution into the experiment. To do this, we collect the equivalent of M experiments where the carrier frequency is incremented over regularly spaced offsets δ_m_ spanning the 2π/TR interval. This is done by keeping the carrier constant and acquiring SSFP series where the phases of consecutive RF pulses are incremented by (Figure 1A):
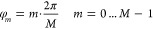
3In the *m*^th^ such experiment, the *S*(0, Φ) signal will accrue a phase that, modulus 2π, is
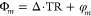
4As per eq 2b, this amounts to acquiring an SSFP series where
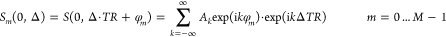
5To obtain frequency selectivity from this array, we propose combining signals as^17^
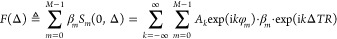
6Here the {*β*_*m*_}_0≤*m*≤*M*-1_ are coefficients of a linear combination built so as to have *F*(Δ) resemble a low-pass filter with a narrow transition width equal to 2π/(TR·NB), with NB being the total number of bands that will fit into the 2π/TR interval. To that end we approximate *F*(Δ) with a low-pass, *N*-point finite impulse response (FIR) filter function *R*(Δ)^20^
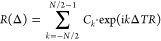
7Since the {*A*_*k*_} in eq 6 decay to zero for large |*k*|, we can equal the demands of eqs 6 and 7, , leading to
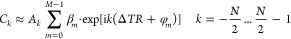
8This relation can be cast in matrix form as
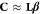
9where **L** is an *N*-by-*M* matrix with elements
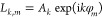
10and **β** is the *M*-by-1 vector being sought for achieving spectral discrimination. This reconstruction problem was solved by minimizing the norm  using a least-squares approach aided by Tikhonov regularization:^21^
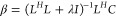
11where **L^H^** is **L**’s conjugate transpose, **I** is an *M*-by-*M* identity matrix, and λ is the regularization parameter.

The **β** coefficients in eq 11 lead to a filter *R*(Δ) centered at zero, whose sharpness will depend on how many {*A*_*k*_} coefficients are significantly different from zero. Maximizing these coefficients requires a relatively small tip angle α (Figure 1C): for a given desired spectral resolution, eqs 1 and 2 enable one to calculate an “optimal” flip angle α_opt_, revealing a spin-isochromat with full intensity if it falls within the band and zero otherwise. Departing from such a value will broaden the band and increase the number of “wiggles” outside it (Figure 1D; see Supporting Information). It is possible to shift this *R*(Δ) filter away from zero and over the remaining NB – 1 spectral bands needed to analyze the full ± π/TR frequency interval, by multiplying the coefficients in eq 8 by j*2π/NB phase-shifts. This leads to a matrix of *N*-by-NB coefficients
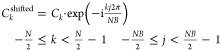
12

This **C** array can be used to solve for the corresponding set of **β** vectors using matrix relations, as further described in the Supporting Information. The result of this will be an *M*-by-NB matrix , whose *j*^th^ column contains the *M* coefficients needed for calculating the spectrum for band *j*. These coefficients will provide a good discrimination provided that the number of phase-incremented SSFP experiments *M* is sufficiently large; in the present study the number *M* of experiments used was set at twice the number of NB bands desired. (Given the acquisition of *M* = 2NB phase-incremented SSFP FIDs, the phase-shifting in the coefficients of eq 12 could in principle also be defined as shifted by half a band—i.e., by π/(NB·TR). The Supporting Information shows how spectral appearance can be improved by solving  and then reconstructing the data twice, with these two sets of **C**-coefficients.

So far the processing introduces spectral resolution but does not address SSFP’s folding problem: peaks separated by multiples of 2π/TR will end up falling on the same band. It is possible to unfold this information by sampling numerous points NP within each TR. Performing a discrete FT (DFT) of these short FIDs will yield an overall spectral width of ≈2πNP/TR, with each data point *p* separated by a frequency increment ≈2π/TR; adding onto this the filtering procedure described in the preceding paragraph can then “dissect” each DFT element into NB finer bands. Although conceptually simple, the quality of this reconstruction will be dependent on the phase correctness of each point in the band-separated  FIDs. These phases will not be uniform but rather affect differently each of the −(NB/2) ≤ *j* ≤ (NB/2) – 1 bands: different bands amount to peaks with different offsets, associated with different first-order phase distortions. In addition, as SSFP leads to a train of echoes peaking at the center of each pulse, dead times will affect the phase contributed by each resonance. To solve these problems, we decoupled the DFT side of the processing from the filter-based processing, by considering the 1D spectral reconstruction as involving a 2D matrix (Scheme 1, top). Neither the NB columns covering a ±π/TR bandwidth nor the NP rows spanning the short FIDs can by themselves describe the full 1D spectrum; however, if unraveled as shown in Scheme 1, they will characterize a total bandwidth ≈2πNP/TR with a resolution ≈2π/NB*TR. In this representation, columns in the  matrix are the bands separated by the -matrix filtering, while rows contain the 1 ≤ *p* ≤ NP FID points for each band:
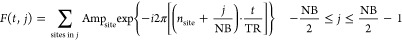
13where *n*_site_ is the integer characterizing a site that has folded onto band *j*. If the SSFP experiment were ideally sampled, peaks belonging to the central *j* = 0 band—i.e., at an offset that is zero or an exact multiple of 2π/TR—would have their evolution phases along *F*(*t*, *j* = 0) beginning and ending in zero. DFT on such a *j* = 0 band will yield sharp spectral peaks, devoid of Gibbs ringing. By contrast, DFT on peaks belonging to *j* ≠ 0 bands for which *F*(*t* = 0, *j*) ≠ *F*(*t* = TR, *j*), will exhibit wiggles after FT, appearing as “sidebands” spaced by 2π/TR after the procedure in Scheme 1. To avoid this, the FID of every band *j ≠ 0* was phase-corrected as , a shearing-like transformation when viewed as operating in a mixed frequency (*j*)/time (*t*) domain (see Supporting Information).

**Scheme 1:**
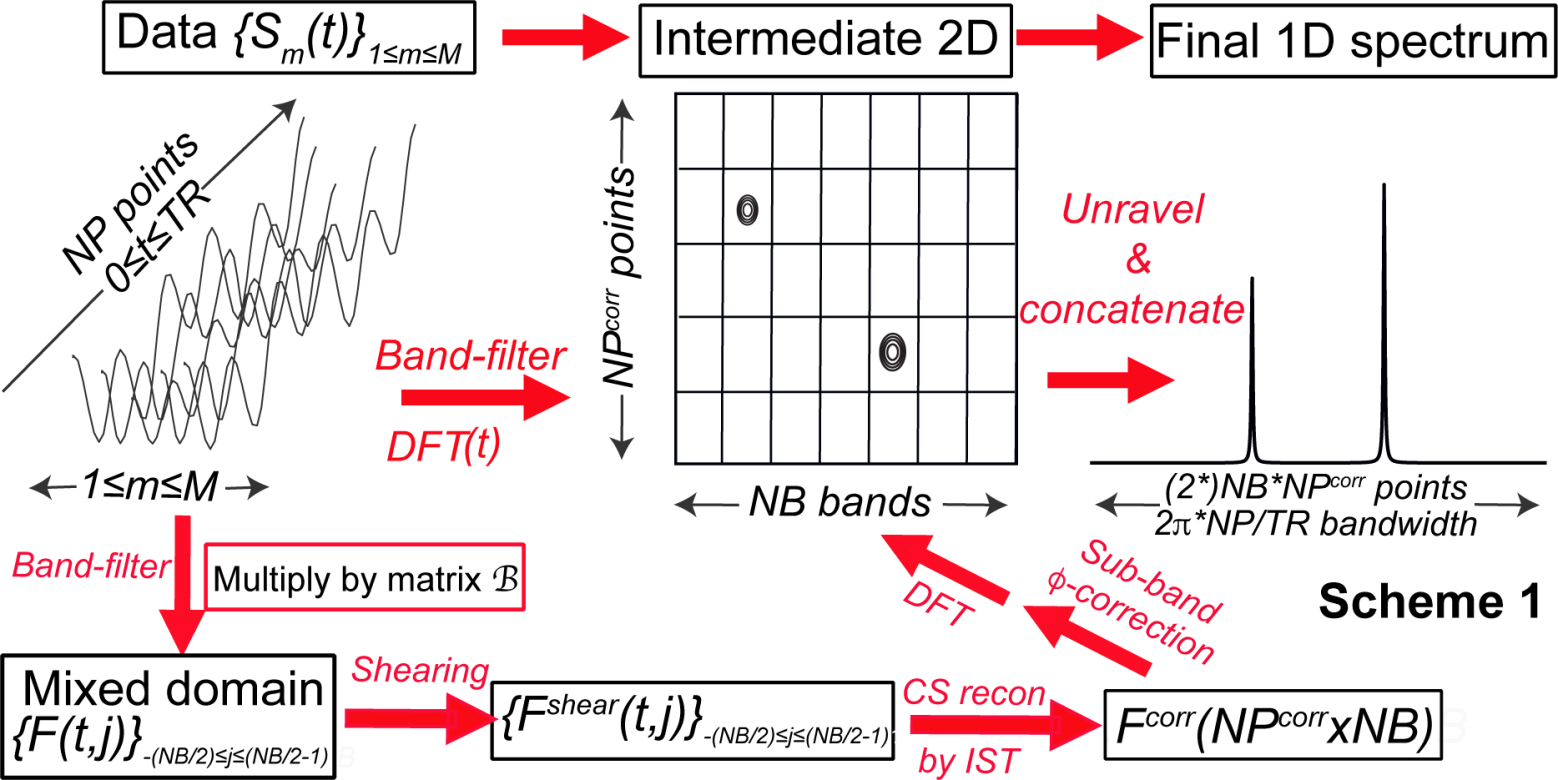
Summary of the Processing Adopted in This Work, Viewing the Collected Data in Two “Dimensions” (Acquisition Time and Phase Increment) Leading to an Intermediate That Is Eventually Unraveled into the 1D Spectrum

Even with this correction, finite pulse widths and limited receiver response times will make *F*(*t* = 0, *j*) ≠ *F*(*t* = TR, *j*). To deal with this, a procedure accounting for the missing points in each band’s FID was developed based on iterative soft thresholding (IST, see Supporting Information).^21,22^ This led to a slightly extended *F*(*t*, *j*) FID set possessing NP^corr^ points, accounting for what should have been acquired over a full TR period. Even this correction did not remove wiggles entirely: an additional phase correction had to be performed to take care of sub-bin shifts. This final step used a 1D version of the 2D subvoxel-shifting introduced by Kellner et al.^23^ With all of this implemented, rearrangement of the NB-separated band spectra along NP^corr^ consecutive intervals provided nearly artifact-free spectra. Notice that as there is no decay in the short FIDs involved in SSFP there are no dispersive components in these spectra, and no resolution is lost by calculating them in magnitude mode. Notice as well that none of these procedures involved any sensitivity enhancements: they are all linear manipulations affecting to the same extent signals and noise.

A series of experimental tests was run to assess the quality of the data arising from this phase-incremented SSFP protocol. Tests focused on high-resolution 1D ^13^C NMR at natural abundance, acquired under both ^1^H decoupling and NOE conditions. The sequence utilized for these acquisitions is described in the Supporting Information, and its performance, although correct, was not perfect as small DC offsets ended up affecting the FIDs. A number of options based on applying “catalytic” pulses^24,25^ to hasten the steady-state conditions based on incremented phases were tested, but provided no visible improvements. These caveats notwithstanding, Figure 2 presents 1D NMR spectra arising using the principles introduced above for glucose and a 1D {^1^H}^13^C FT-NMR spectrum collected under optimized conditions. The quality of the two data sets is comparable, although peak intensities are not identical as in each set relaxation weights-in differently. Still, even when the resonances are closely spaced (e.g., the C5 resonances of the α and β anomers in Figure 2, separated by 0.06 ppm), the SSFP approach manages to resolve the peaks. Notice that this separation benefits from optimized flip angles α_opt_; larger flip angles and/or fewer phase increments yielded, as expected, a decrease in resolution. While the resolution of the FT-NMR spectrum appears superior to that of the SSFP variants here assayed, the SNR_t_ for some of the peaks appeared equal to or better for the SSFP variants.

**Figure 2:**
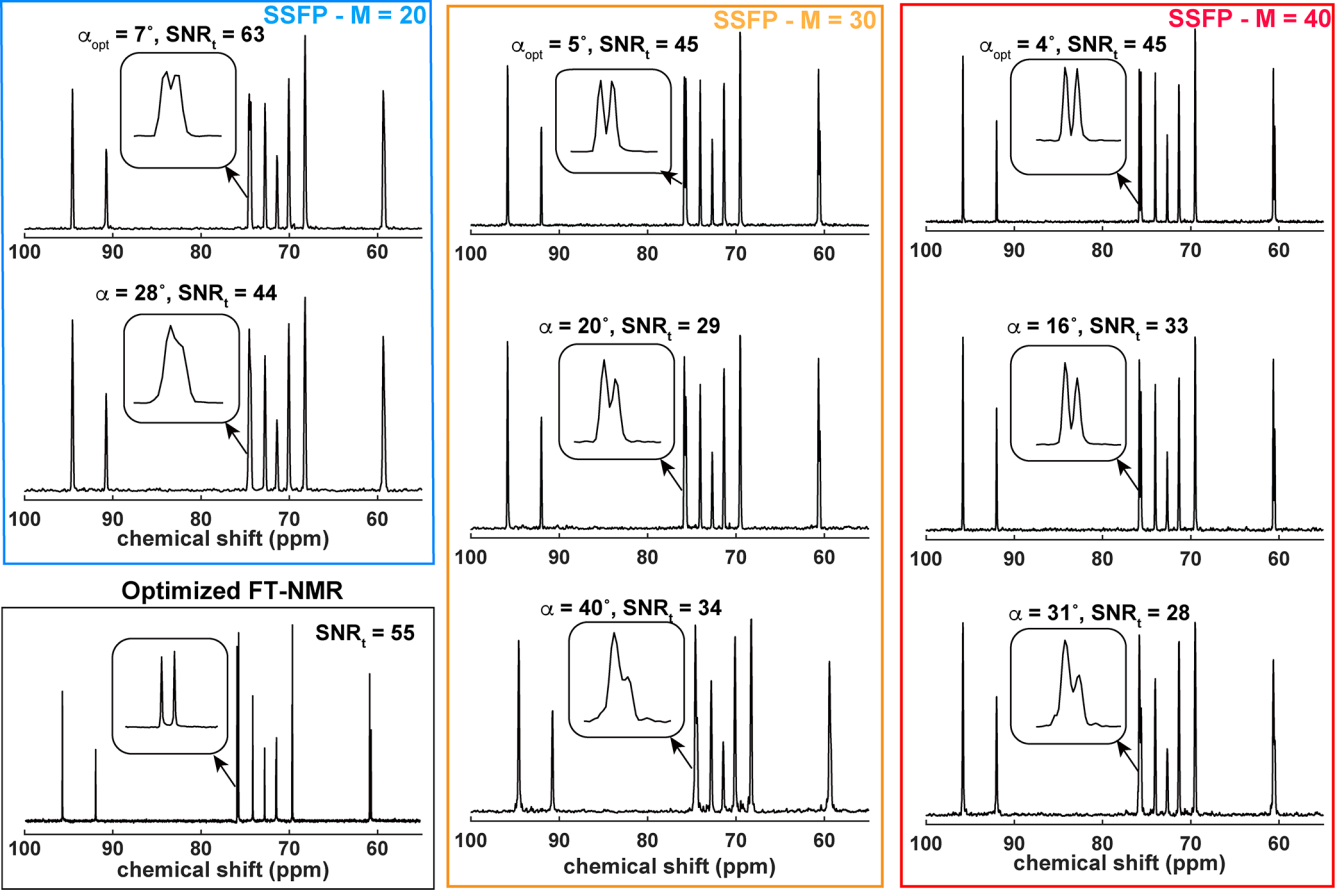
^13^C NMR spectra of 50 mM glucose in D_2_O recorded using FT-NMR (Ernst-angle excitation, 1 s acquisition) and the SSFP-based strategy for different pulse angles and number of phase increments. In these cases, L1 = 1000 pulses spaced by 5 ms and a spectral width = 200 kHz were collected, nested in the indicated *M* phase-loops. Shown for each experiment is the SNR_t_, where SNR was calculated solely for the strongest peak and noise arose from the >140 ppm range. Insets show the resolution achieved by each experiment for the α and β C5 carbons. All experiments were run with constant ^1^H irradiation; see the text for further details.

Figure 3 presents a similar analysis of {^1^H}^13^C NMR cholesterol spectra. Once again, suitable choices of the phase increments provide SSFP with a resolution comparable to that of FT-NMR. The resolution-dependence on the flip angle is also evident, while the sensitivity of the experiment is largely independent of this α and compares well with FT-NMR acquisitions.

**Figure 3:**
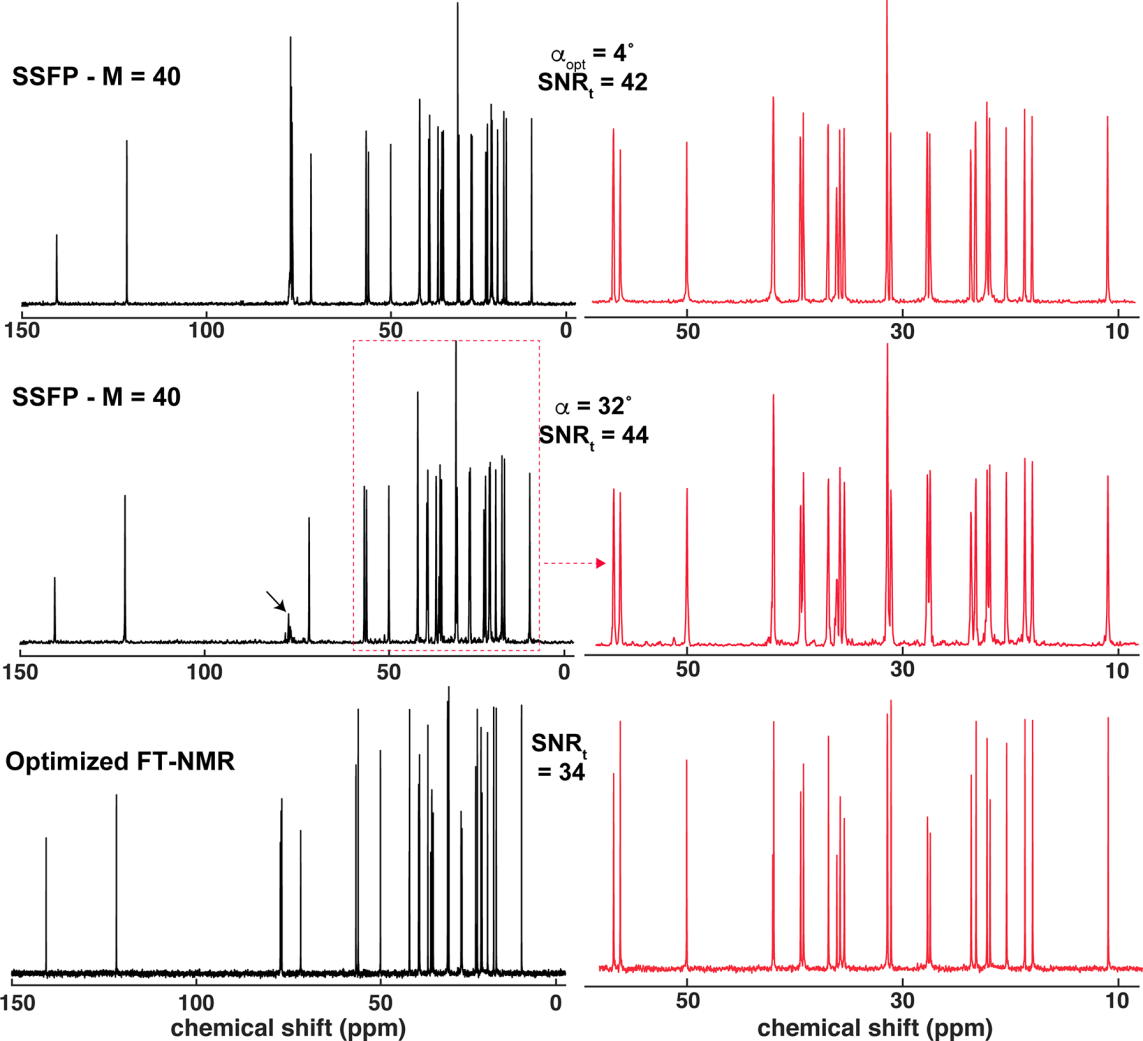
1D ^13^C NMR spectra of 50 mM cholesterol in CDCl_3_ recorded as described in Figure 2; SSFP are only shown for *M* = 40 using two flip angles. Spectra in red, corresponding to a zoomed 8–55 ppm region, illustrate the resolution of the various traces. Distortions in the chloroform peak (arrow, centered close to the spectral center) stemmed from our use of baseline correction. See the text for further details.

The present study introduced an alternative to FT-NMR that, based on SSFP, can deliver customary-looking 1D spectra. SSFP was assessed as its SNR_t_ advantages when dealing with a single resonance have long been known in MRI;^11−15^ they have also been demonstrated in spectroscopic imaging applications involving a small number of resonances,^26−28^ and hold for certain static and spinning solid NMR experiments.^29^ In the present high-resolution case, a very wide set of parameters could, in principle, be explored for comparing the SNR_t_ of FT- and SSFP-based experiments. Furthermore, FT-NMR has developed over the years an arsenal of well-understood tools for maximizing SNR_t_, which remains to be explored for the SSFP case. Indeed, alternative acquisition and processing avenues can be conceived for the latter, that have no parallel in FT-NMR. It also remains to be seen if related approaches can be devised when dealing with *J*-couplings, as well as with multidimensional acquisitions. These aspects will be discussed in upcoming studies.

## Supporting Information Available

Materials and methods used, pulse sequence, details about the band-selective filter design and the spectral binning, provisions taken to account for various sources of spectral Gibbs ringing, improvement of the spectral appearance by phase-shifting the filters, and summary of the data processing procedure (PDF)

## Abbreviations

DFT: discrete Fourier transform
FID: free induction decay
FT: Fourier transform
SNR: signal-to-noise ratio
SNR_t_: SNR/√(acquisition_time)
SSFP: steady-state free precession
