# Supplementary material for: Phase-Incremented Steady-State Free Precession as an Alternate Route to High-Resolution NMR
Source: J Am Chem Soc. 2024 Jan 31;146(6):3615–21. doi: 10.1021/jacs.3c12954 (PMC10870713; doi:10.1021/jacs.3c12954)
Supplement: Supplementary file 1 — ja3c12954_si_001.pdf [file ja3c12954_si_001.pdf]

Supporting Information for

Phase-incremented Steady State Free Precession as an Alternate Route to High Resolution NMR

Tian He,[1,2] Yuval Zur,[3,*] Elton T. Montrazi,[1] and Lucio Frydman[1,*]

[1] Department of Chemical and Biological Physics, Weizmann Institute, 7610001 Rehovot, Israel; [2]Department of Chemistry, Zhejiang University, Hangzhou 310058, P. R. China; [3]Insightec Ltd, 3903203 Tirat Carmel, Israel

*Emails: yuvalzur50@gmail.com; lucio.frydman@weizmann.ac.il

Materials and methods

All samples investigated in this study were purchased from Sigma/Aldrich and used as received. NMR experiments were performed on a Bruker 600 MHz spectrometer using an AVIII HD console running Topspin 3.2, equipped with a TCI Prodigy® probe. The SSFP pulse sequence was written on the basis of two nested loops, whereby a train of M α_{φ_m}-FID acquisition sets, possessing flip angles α and having their RF phases serially incremented by $\{\varphi_m = (m-1)\cdot\Delta\varphi\}_{1\leq m\leq L2}$, were looped L2 times while incrementing their $\Delta\varphi$'s as $\Delta\varphi_k = (k-1)\cdot\frac{360^\circ}{M}$. As the interpulse time TR was only a few milliseconds, this was repeated ceaselessly L1 times for the sake of signal averaging, and the ensuing signals were coadded. While the sequence should have benefited from conventional 0/180° phase cycling, such procedure still left a residual DC offset; thus FIDs were corrected post-acquisition. "Catalysis" pulse schemes helping SSFP to reach a steady state upon changing $\Delta\varphi_k$ were assayed, but failed to give significant SNR_t improvements and their results are thus not presented. Given that under the operating $TR \ll T_2, T_1$ conditions SSFP will create echoes that will form at the beginning and end of each TR-long acquisition (in fact, centered symmetrically on consecutive pulses), care was taken to minimize the number of points that were lost due to pulse width and receiver deadtime effects. This was done by working using short (≤10 μs) pulses and large (40-200 kHz) receiver bandwidths while collecting close to the maximum number (524k) of sampled points that the spectrometer could accommodate; none of these choices incurred in any penalties SNR-wise. All ^{13}C experiments using continuous GARP-based heteronuclear ^1H decoupling.

SNR was in all cases calculated as the maximum of the signal divided by the standard deviation of the noise. SSFP sequences were simulated using a Matlab-based Bloch simulator, taking into account T_1 and T_2 relaxation but devoid of J-couplings effects.

Additional data processing considerations

Realizing the band-selective filters. As mentioned in the main text, processing the phase-incremented SSFP data involves two complementary steps: the filter-based separation of peaks within NB bands spanning a ±π/TR frequency interval, and the unfolding of peaks within each of these bands by a DFT of the points collected within each TR. The filter-based separation is done by collecting a series of phase-incremented steady-state scans, with M phases equally spaced around the unit circle:

$$\varphi_m = m \cdot \frac{2\pi}{M}, \ \ m = 0 \ to \ M-1 \qquad [S1]$$

This leads to an $\{S_m\}, \ m = 0 \ldots M-1$ set of FIDs, from which selectivity is created by a linear combination of the scans. Based on Eqs. [7] and [8], this will be given by

$$R(\Delta) \approx \sum_k \sum_m A_k \exp\left(ik\varphi_m\right) \cdot \beta_m \cdot exp(ik\Delta TR), \qquad [S2]$$

where β_m are the coefficients of the linear combination.

We can design an N-point *R(Δ)* filter function using design programs, such as **firpm** of **firls** in the MATLAB signal processing toolbox; e.g.,

$$R(\Delta) = \sum_{k=-N/2}^{\frac{N}{2}-1} C_k \cdot \exp(ik\Delta TR) \qquad [S3]$$

where C_k are the points of the filter's impulse response. From Eqs. [S2] and [S3] it follows that:

$$C_k \approx A_k \sum_m \beta_m \exp(\mathrm{ik}\varphi_m) \qquad [S4]$$

or in matrix form

$$\boldsymbol{C} \approx \boldsymbol{L} \cdot \boldsymbol{\beta} \qquad [S5]$$

Here L is a N-by-M matrix whose element *k,m* is:

$$L_{k,m} = A_k \exp(\mathrm{ik}\varphi_m), \qquad [S6]$$

and $\boldsymbol{\beta}$ is a M-by-1 vector whose coefficients were estimated by minimizing the norm $\| \boldsymbol{C} - \boldsymbol{L\beta} \|$.

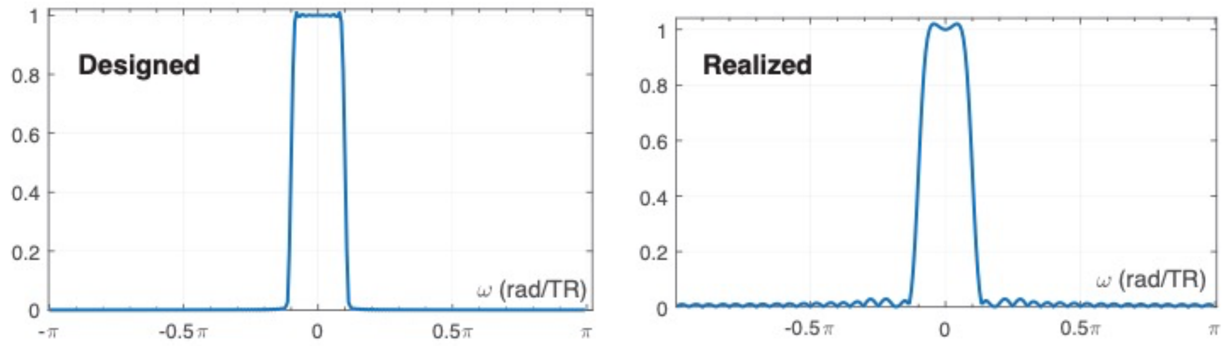

Figure S1: Comparison between a FIR lowpass filter F(Δ) designed with N = 150 points and cutoff frequency of ±π/(20·TR) (**left**), and the approximate filter R(Δ) resulting from a linear combination of M = 20 phase-incremented SSFP scans with RF flip α = 3.9° (**right**).

Figure S1 shows the difference between an ideal FIR low-pass filter designed with N = 150 points and cutoff frequency of ±π/(20·TR), and the filter arising from M = 20 phase-incremented SSFP experiments for TR = 5 msec, T1/T2 = 1000/500 msec and an RF flip angle α = 3.9°. Figure S2 shows another aspect of this calculation, depicting the residual norm of $\| \boldsymbol{C} - \boldsymbol{L\beta} \|$ vs. the RF flip angle α. Notice that the norm is a minimum at 3.9°, which is what we denominate the "optimal" α_{opt}. Notice as well the relatively shallow dependence of the residual on larger flip angle.

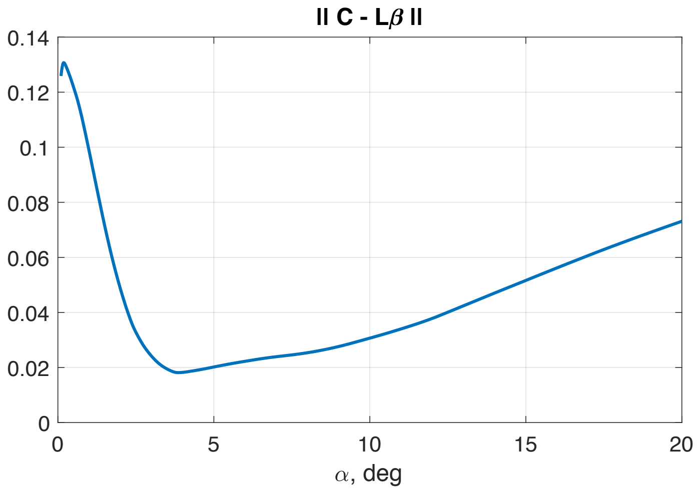

Figure S2: Plotting the norm $\| \boldsymbol{C} - \boldsymbol{L\beta} \|$ vs. RF flip angle α for the conditions depicted in Figure S1. The minimum is at the optimal α_{opt} = 3.9°.

The filters in Figure S1 are centered at zero frequency. To create a group of NB filters of width 2π/(TR·NB) each, the kth point C_k of the impulse response was multiplied by $exp\left(-ikj\frac{2\pi}{NB}\right)$, Eq. [12]. This leads to NB shifted vectors **C**, each of which is to be solved by minimizing $\| \boldsymbol{C} - \boldsymbol{L\beta} \|$. To make this solution more compact, all the **C** vectors were arranged into a single N-by-NB matrix $\boldsymbol{\mathcal{D}}$ where the jth column in $\boldsymbol{\mathcal{D}}$ is the original filter shifted by $j\frac{2\pi}{NB}$. The matrix $\boldsymbol{\mathcal{D}}$ can replace in the treatment above the vector **C**. This result in an M-by-NB

matrix $\boldsymbol{\mathcal{B}}$ corresponding to the coefficients that will filter out a band of width 2π/TR·NB radians/s, centered at an angular frequency offset $j\frac{2\pi}{NB\cdot TR}$. These were once again solved by a least-square, regularized approach

$$\boldsymbol{\mathcal{B}} = (\boldsymbol{L^H}\,\boldsymbol{L} + \lambda \boldsymbol{I})^{-1}\boldsymbol{L^H}\boldsymbol{\mathcal{D}} \qquad [S7]$$

Accounting for Gibbs ringing upon DFT. 1 – The shearing transformation

Another challenge that these experiments need to deal with, concerns the phase distortions that arise upon processing the short FIDs in Eq. [13]. Looking at a specific band *j,* the ensuing 0 < t < TR signal will, for a single isochromat of frequency ν_j, be

$$F(t,j) = Amp_j\ \exp\left(-i2\pi\nu_j t\right) \qquad [S8]$$

where we have disregarded the T_2 >> TR decay as well as any constant phase factors, and where

$$\nu_j = \frac{1}{TR}\cdot\left(n + \frac{j}{NB}\right) \qquad [S9]$$

and so

$$F(t,j) = Amp_j \exp\left\{-i2\pi[(n + \frac{j}{NB})\cdot\frac{t}{TR}]\right\} \qquad [S10]$$

Figure S3 shows the consequences of performing a DFT on *F(t, j≠0)*, without accounting first for the phase distortion arising from a band's $\frac{j}{NB\cdot TR}$ offset effect. Notice the strong ringing for this site with n = 10 and j = NB/2, appears spilling in neighboring n-values (Fig. S3, left); these

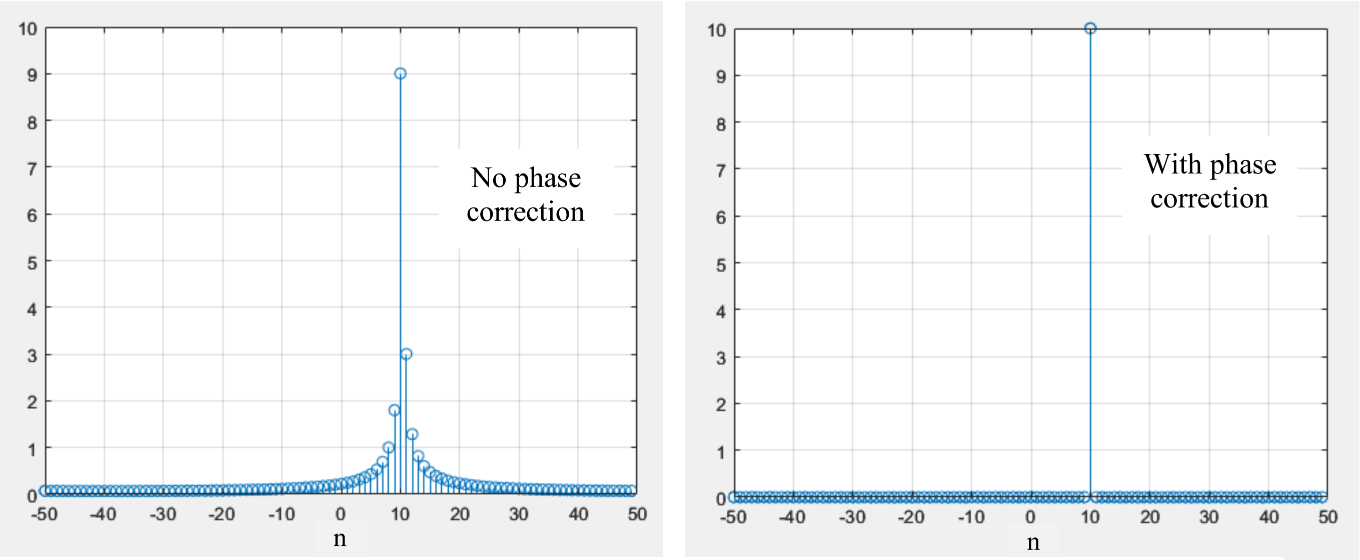

Figure S3: Removing the "wiggles" associated to phase-correction problems upon doing a discrete FT on the truncated FIDs collected in the SSFP experiments. The -50≤*n*≤*50* points span the entire width of the final spectrum, which in our experiments ranged between 40 and 200 kHz. The truncation wiggles thus appear as "sidebands" of the legitimate resonances, degrading the apparent SNR of the final trace.

will look as "sidebands" in the fully unraveled 1D reconstructed spectrum (Scheme 1). On the other hand, by applying a shearing-like phase correction proportional to t and j,

$$F^{shear}(t,j) = F(t,j) \cdot \exp\left(i2\pi \frac{j}{NB}\frac{t}{TR}\right), \quad [S11]$$

$F^{shear}(t=0,j)$ becomes equal to $F^{shear}(t=TR,j)$ for any j, and a single peak be obtained upon DFT (Fig. S3, right).

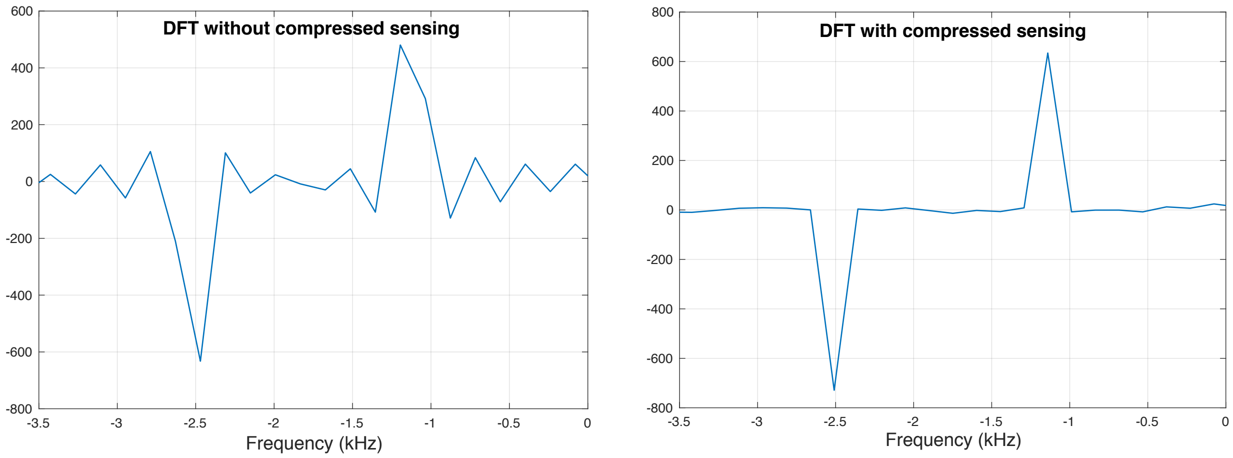

Figure S4: Removing "wiggles" associated to an incomplete sampling of the FID over the $0 \leq t \leq TR$ interval. The data corresponds to band 17 of the glucose spectrum (Figure 2, left-hand column; NB=20), containing the two peaks in the 60-70 ppm region. As the FID of this band was sampled with 25 µsec dwell times and TR was 6.6 ms it should have contained 264 points, but only 252 could be faithfully sampled. DFT of these data leads to strong Gibbs ringing **(left)**, even after the correction in Eq. [11]. Applying a compressed sensing reconstruction completes the missing points, reinstates the periodicity of the FID, and leads to a DFT with much weaker ringing (**right**).

Accounting for Gibbs ringing upon DFT. 2 – Completing missing FID points and performing a sub-bin phase correction

Eq. [S11] often fails to perform a perfect phase correction, for two reasons. One of these relates to the impossibility to sample every data spanning the t = 0 to t = TR time interval. In an actual experiments points will be lost due to finite RF pulse widths, finite filter response times and other factors. This leads again to $F^{shear}(t=0,j) \neq F^{shear}(t=TR,j)$, causing additional Gibbs ringing upon DFT vs t. Eliminating this unwanted ringing requires estimating the missing data points on the basis of the data that were collected; in other words, start from $F^{shear}(t)$, and estimate for it the missing points for a corrected $F^{corr}(t)$ FID spanning from 0 to TR. To do so we rely on the fact that, if these correcting FID points are found, the spectrum arising upon DFT$\{F^{corr}(t)\}$, will be sparser than that arising from DFT$\{F^{shear}(t)$. This additional sparsity will derive from the elimination of the ringing, as illustrated in Figure S4 utilizing glucose data from the experiment shown in Figure 2. To estimate $F^{corr}(t)$ from $F^{shear}(t)$ we use an iterative soft thresholding (IST) algorithm based on compressed sensing,[22]

that increases the number of time-domain points from NP to NP^{corr}, while relying on the sampled points in $F^{shear}(t)$ to calculate the missing data points while minimizing the first norm of the DFT$\{F^{corr}(t)\}$.[30] This algorithm promotes a sparse solution which is consistent with the collected data, is stable, converges after 5-10 iterations, and leads to significantly reduced/eliminated Gibbs ringing (Figure S4). We applied this algorithm to all the NB columns of $F^{shear}(t, j)$, leading to an improved matrix F^{corr} of NP^{corr} rows and NB columns.

Even with the aforementioned corrections, spectral lines may fail to oscillate by an exact integer number n of cycles between t = 0 and t = TR. This is because spectral lines may fall at frequencies that are not integer multiples of $\frac{2\pi}{TR \cdot NB}$, and hence the shearing correction in Eq. (S11) will leave a small phase distortion even after its application. To eliminate the remaining Gibbs ringing we applied the method of Kellner *et al* to each column of $F^{corr}(t, j)$ – which is equivalent to making a sub-dwell phase correction.[23] This correction is once again based on minimizing the Gibbs ringing arising upon DFT of the phase-corrected vector. This time, a frequency-dependent sub-pixel phase correction is applied, within a range of shifts ranging from -0.5 to 0.5 pixels. The phase correction that minimizes the Gibbs ringing around a given pixel, is then applied to that particular pixel. Supporting Figure S5 demonstrates the algorithm in action, by considering the signal of 3 sites precessing with an arbitrary, non-integer number of cycles in the sampling window. The DFT of the uncorrected signal (left-hand panel) shows a significant Gibbs ringing; the spectrum after Kellner's correction (right-hand panel) has significantly reduced ringing.

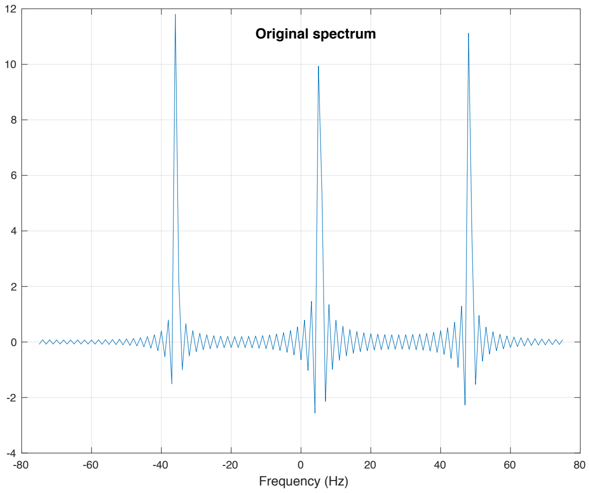

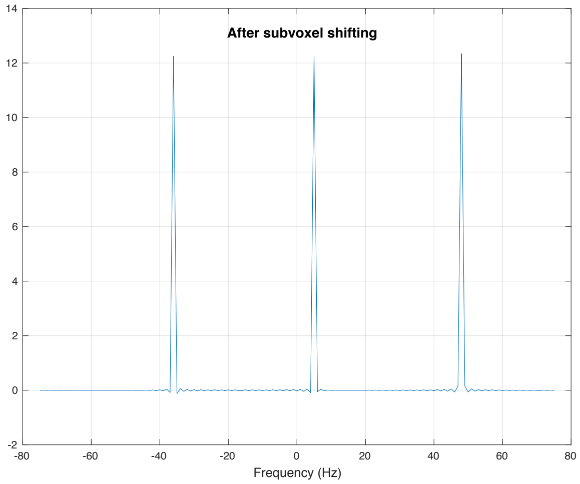

Figure S5: (Left) DFT of three sites precessing without decay with equal amplitudes at -35.85, 5.35 and 48.25 Hz. Since the spectral window was sampled at integer multiples of 1 Hz, the number of cycles of the sites in the FID is not an integer and there is significant Gibbs ringing. **(Right)** Spectrum arising after applying Kellner's algorithm, the ringing is significantly reduced.

Achieving a finer spectral binning.

In the band-filtering treatment above, it was assumed that each spectral line is fully contained within a single bin of frequency $\nu_j = j\frac{2\pi}{NB\cdot TR}$. However, it is possible that the spectral line is equally shared between two adjacent bins, j and j + 1; in that case the signal amplitude appearing in bins j and j + 1 will be half the true value, and the spectral width will be twice as wide. This is demonstrated in Figure S6a, that shows a single row from an ideal *F(t=0,j)* matrix, assumed to possess two spectral lines with equal width and equal amplitude within an NB=15 bin spectrum. The first spectral line is fully contained in bin 5, whereas the second line is equally shared by bins 11 and 12; by missing being centered at a bin that is at an integer multiple of $\frac{2\pi}{NB\cdot TR}$, the amplitude of the latter is halved and its signal is twice as wide. One could solve this by computing a second set of NB filters, similar to the original one but shifted by half a bin width; i.e., by $\frac{\pi}{NB\cdot TR}$. With these new filters it is now the first spectral line (originally at j = 5) that is partly located in two neighboring bins, while the second spectral line (originally at j = 11.5) is now fully contained in a bin of its own (Figure S6b). The information in these two spectra can be used to restore a higher resolution spectral set, with each peak containing a corrected amplitude and width. To do so we denote the signals in Figures S6a and S6b as S1 and S2; a correction is then done by: 1) Interpolating (linearly) S1 and S2 by a factor of 2; since in this case S1 and S2 had 15 bins, the interpolated data, S3 and S4, will span 30 bins. 2) One of these data sets (e.g. S4) is shifted by one bin, since its filters were shifted by half of the original bin. 3) S3 and S4 are co-added to produce a new output signal S_out with an NB=30 bin spectrum, as shown in Figure S6c. S_out restores the amplitude of the lines and makes their spectral width the same, even if the Full-Width at Half-Maximum (FWHM) of the lines in S_out is ca. 30% wider than the original lines when they were fully contained in a single bin. The advantage, however is that one does not have to worry now about the location of the spectral lines with respect to the bins. This procedure was adopted throughout this study.

Summary.

To sum up, the main steps involved in the acquisition and processing of phase-incremented SSFP spectra comprise:

1) Acquiring an array of data sets $\{S_m(t)\}_{1\le m\le M}$ containing $1 \le p \le NP$ time-domain points and M phase-incremented SSFP scans. As TR in these SSFP acquisitions is very short (≈5ms), hundreds or thousands of such FIDs may be collected within a few seconds; these are co-added for the sake of SNR improvement.

2) Calling the resulting data matrix $S = \{S_{p,m}\}_{1\le m\le M, 1\le p\le NP}$, these data are subdivided into a series of NB bands by multiplying $\mathcal{S}$ by a $\mathcal{B}$-matrix that separates peaks in NB bins within a severely folded ±π/TR frequency range.

3) The ensuing 2D $F(t,j) = \{F_{p,j}\}_{-\frac{NB}{2}\le j\le\frac{NB}{2}-1, 1\le p\le NP}$ mixed frequency/time-domain data set is phase-corrected by a term-by-term multiplication with a "shearing" matrix $\boldsymbol{\mathcal{P}} = \exp\left(i\frac{2\pi j}{NB}\frac{t}{TR}\right)$.

4) A DFT of each of the columns in this matrix unfolds the peaks that were overlapping within each band. To remove residual Gibbs ringing effects affecting the peaks, a combination of compressed sensing and sub-bin shifting corrections is applied.

5) The rows in the resulting 2D frequency/frequency matrix are concatenated into a single one-dimensional spectrum.

6) The whole procedure is repeated with the filters in the $\mathcal{B}$-matrix shifted by half a bin, and the results are suitably coadded into a single final trace, whose magnitude plot leads to the desired spectrum.

Though apparently involved, this process is fully automated and takes a second or so on a laptop computer.

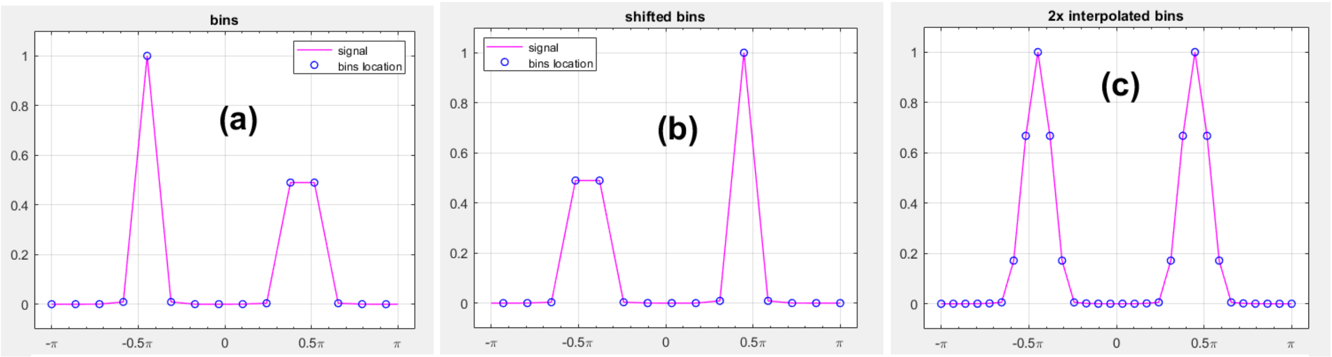

Figure S6: Processing a row from the F-matrix with NB = 15 bands, having two spectral lines with equal amplitude and width located at bands 5 and 11.5 respectively. **(a)** Signal arising at each filter bin: the line at bin 11.5 shows lower and broader. **(b)** Same but with filters shifted by half a bin; now the line originally at bin 5 is lower and broader. **(c)** Restoring the information from (a) and (b) by coadding both spectral sets after their linear interpolation into 30 bins. The width of the lines is 30% larger than the width of the line that fits into a single bin in (a) and (b), but the resulting trace is more faithful to the real peak shape.
